# An Overview of Some Biopesticides and Their Importance in Plant Protection for Commercial Acceptance

**DOI:** 10.3390/plants10061185

**Published:** 2021-06-10

**Authors:** Jitendra Kumar, Ayyagari Ramlal, Dharmendra Mallick, Vachaspati Mishra

**Affiliations:** 1 Bangalore Bioinnovation Centre (BBC), Life Sciences Park, Electronics City Phase 1, Bengaluru, Karnataka 560100, India; director@bioinnovationcentre.com; 2 Division of Genetics, Indian Agricultural Research Institute (IARI), Pusa Campus, New Delhi 110012, India; ramlal.ayyagari@gmail.com; 3 Department of Botany, Deshbandhu College, University of Delhi, Delhi 110019, India; dkmallick@db.du.ac.in; 4 Department of Botany, Dyal Singh College, University of Delhi, Delhi 110003, India

**Keywords:** biopesticides, agriculture, food supply, microorganisms

## Abstract

Biopesticides are natural, biologically occurring compounds that are used to control various agricultural pests infesting plants in forests, gardens, farmlands, etc. There are different types of biopesticides that have been developed from various sources. This paper underscores the utility of biocontrol agents composed of microorganisms including bacteria, cyanobacteria, and microalgae, plant-based compounds, and recently applied RNAi-based technology. These techniques are described and suggestions are made for their application in modern agricultural practices for managing crop yield losses due to pest infestation. Biopesticides have several advantages over their chemical counterparts and are expected to occupy a large share of the market in the coming period.

## 1. Introduction

The global population is exploding at an exponential rate and is anticipated to reach approximately 9.7 billion by 2050, the largest share of which is in Africa and Asia [1]. This has imposed a large burden on agriculture and its allied sectors in terms of meeting food demands, which requires more inputs for crop production. Anthropogenic activities have affected people’s surroundings and have also had negative impacts on the environment and ecosystems, including reductions in agricultural areas due to construction, the explosion of nutrient mining, degradation, and contamination of water resources (resulting in scarcity), aggregation of xenobiotics in the soils, and degeneration and deterioration of the quality, fertility, and efficiency of soil, with implications of soil erosion and climate change. In order to overcome these challenges and meet the requirements for food and supplies, the productivity and sustainability of agricultural practices should be improved and novel and improved strategies must be found. Enhanced agricultural productivity can be achieved in many ways, such as through increasing crop yield by providing manure and organic-based treatments, including biopesticides, or by limiting yield loss due to extreme environmental conditions (such as biotic and abiotic stresses) [2,3]. Abiotic stress can be largely controlled by the use of biostimulants and bioeffectors [4]. Biopesticides, which are pest management agents based on living microorganisms or natural products, offer a great promise in controlling yield loss without compromising the quality of the product.

The chemical pesticides used in crop protection, to reduce the damage caused by pathogens and pests in agricultural fields, pose many long-term threats and risks to living beings due to their harmful side effects. They are known to cause cancers [5] and foetal impairments [6] and they persist in the environment for many years (i.e., they are nonbiodegradable) [7]. Furthermore, based on their potential application and strong inhibitory activity against pests, these synthetic pesticides dominate the market and have a significant impact on the manufacture of products [8]. Based on a report by Business Communications Company (BCC), Inc., research on the global biopesticide and synthetic pesticide market showed that it was worth USD 61.2 billion in 2017 and is expected to rise to approximately USD 79.3 billion by 2022 [9,10]. Nutrient reduction and an increased disease incidence are quite common in crops grown on soils heavily subjected to chemical pesticides [11], and this is undesirable from the agricultural soil management for food and nutritional security standpoint. According to the Food and Agriculture Organization (FAO), United Nations (2017–2018), the top three leading pesticide-consuming countries are China, the USA, and Brazil [12]. In addition, pesticide consumption in India drastically increased from 50,410 tonnes (T) in 2016 to 58,160 T in 2018 [13]. The pesticides utilised for crops are as follows: fibre crops account for around 67%, fruits 50%, vegetables 46%, spices 43%, oilseeds 28%, and pulses 23% [14,15]. According to an annual report by the Ministry of Chemicals and Fertilizers, India (MoCF) (2019–2020), the production or manufacture of chemical pesticides increased from 186,000 metric tons (MT) in 2014–2015 to 217,000 MT in 2018–2019 [15]. The FAO also reported that from 2015 to 2018, the share of global pesticide consumption was 52.2% in Asia, 32.4% in the USA, 11.8% in Europe, 2% in Africa, and 1.6% in Oceania [12]. The per hectare consumption of pesticides by country is highest for China, followed by the UK, with the least in India [12]. Of the Indian states, Jammu and Kashmir had the highest chemical pesticide consumption, followed by Andhra Pradesh [13,14]. Based on the statistics of chemical pesticide consumption in India alone, it is imperative to seek alternative methods, especially to increase the use of biopesticides [15].

Biopesticides are naturally occurring compounds or agents that are obtained from animals, plants, and microorganisms such as bacteria, cyanobacteria, and microalgae and are used to control agricultural pests and pathogens. According to the US Environmental Protection Agency, biopesticides are ‘derived from natural materials such as animals, plants, bacteria and certain minerals’ [16]. Products such as genes or metabolites from these biocontrol agents can be used to prevent crop damage [16]. The use of biopesticides is, by far, more advantageous than the use of their counterparts, traditional chemical pesticides, as they are eco-friendly and host specific [17]. The use and application of agro-based chemicals in the agricultural sector to protect crop plants from invading and infecting pests can be greatly improved by employing biopesticides [17,18].

This paper provides up-to-date information on various important biopesticides, including types, advantages, and their utility in plant protection that would eventually lead to their commercial acceptance. Furthermore, various potential sources and technology involved in the production of biopesticides are briefly described.

## 2. Types of Biopesticides

There are many types of biopesticides, and they are classified according to their extraction sources and the type of molecule/compound used for their preparation [19]. The categories are listed below.

### 2.1. Microbial Pesticides

These are derived from microorganisms including bacteria, fungi, and viruses. The active molecules/compounds isolated from these organisms attack specific pest species or entomopathogenic nematodes. Those known as bioinsecticides, target insects that harm crops, while those that control weeds via microorganisms, such as fungi are referred to as bioherbicides. Over the last decade, extensive research activities on microbial biopesticides have led to the discovery and development of a good number of biopesticides and have paved the way for their marketability [19]. The successful use of *Bacillus thuringiensis* (Bt) and some other microbial species led to the discovery of many new microbial species and strains, and their valuable toxins and virulence factors that could be a boon for the biopesticide industry, and some of these have been translated into commercial products as well [19,20]. Major groups of bacterial entomopathogens include species of *Pseudomonas*, *Yersinia*, *Chromobacterium*, etc., while fungi comprise species of *Beauveria*, *Metarhizium*, *Verticillium*, *Lecanicillium*, *Hirsutella*, *Paecilomyces*, etc. [18,21]. Other important microbial pesticide producers are baculoviruses that are species specific and their infectivity is associated with the crystalline occlusion bodies that are active against chewing insects (Lepidopteran caterpillars) [18]. The baculoviral occlusion body is basically a virion that is combined with the Bt toxin to produce recombinant baculovirus (ColorBtrus), producing occlusion bodies that incorporate the Bt insecticidal Cry1Ac toxin protein for enhancing the speed of action and pathogenicity with respect to its wild-type counterpart [18]. Entomopathogenic nematodes (EPNs) used as biocontrol agents belong primarily to species in the genera *Heterorhabditis* and *Steinernema*, associated with mutualistic symbiotic bacteria of the genera *Photorhabdus* and *Xenorhabdus* and are safe to mammals, environment, and nontarget organisms [18]. Their commercial development as biocontrol agents has been convenient because of their ease in mass production, using in vivo or in vitro techniques, and exemption from registration [18].

### 2.2. Biochemical Pesticides

Biochemical pesticides are naturally occurring products that are used to control pests through nontoxic mechanisms, whereas chemical pesticides use synthetic molecules that directly kill pests. Biochemical pesticides are further classified into different types depending upon whether they function in controlling infestations of insect pests by exploiting pheromones (semiochemicals), plant extracts/oils, or natural insect growth regulators.

#### 2.2.1. Insect Pheromones

These are chemicals produced by insects which are mimicked for use in controlling insects in the integrated pest management programs. These chemicals are effective in disrupting insect mating to prevent the success of mating, thus reducing the number of insect progeny. The insects exploited in this process act as dispensers of pheromones that become confused due to the presence of pheromone flumes diffused in the surroundings. Insect pheromones are not true ‘insecticides’ since they do not kill insects but influence their olfactory system to affect behaviour [22]. A detailed account of the mode of action of pheromones is given by Ujváry [20]. In summary, the antennae of the perceiving insect adsorb pheromones, which then diffuse into the interior of the sensilla through microscopic pores in the cuticle. Once inside, these are transferred through the hydrophilic sensillum to the chemosensory membranes by pheromone-binding proteins (PBPs). Subsequently, the pheromone or pheromone–PBP complex interacts with a specific receptor protein, which transduces the chemical signal into an amplified electric signal by a second messenger system connected with neuronal machinery [23].

#### 2.2.2. Plant-Based Extracts and Essential Oils

Over the last several years, plant-based extracts and essential oils have emerged as attractive alternatives to synthetic insecticides for insect pest management. These insecticides are naturally occurring insecticides as they are derived from plants and contain a range of bioactive chemicals [24]. Depending on physiological characteristics of insect species as well as the type of plant, plant extracts and essential oils (EOs) exhibit a wide range of action against insects: they can act as repellents, attractants, or antifeedants; they also may inhibit respiration, hamper the identification of host plants by insects, inhibit oviposition and decrease adult emergence by ovicidal and larvicidal effects [25,26,27]. Their composition varies greatly. Well-known examples in this regard are neem and lemongrass oil, which are very common in global herbal markets. A comprehensive study by Halder et al. [26] showed that a combination of neem oil with entomopathogenic microorganisms, including *Beauveria bassiana*, was very successful against vegetable sucking pests. However, it is very important to determine the dose of azadirachtin content in neem oil so as not to kill the nontarget organisms [28]. A similar strategy has to be established for the entomopathogenic fungi that need to be supported by complementary laboratory bioassays, station, and/or field experiments for effective management of the target pests without affecting nontarget insects [29]. As regards the marketability of essential oils, they in fact, represent a market estimated at USD 700.00 million and a total world production of 45,000 tons, and industries in the US are able to bring essential oil-based pesticides to market in a shortened time period, as compared to the time taken in conventional pesticide launch [30].

#### 2.2.3. Insect Growth Regulators

Insect growth regulators (IGRs) inhibit certain fundamental processes required for the survival of insects, thereby killing them. Furthermore, these compounds are highly selective and less toxic to nontarget organisms [23]. Depending on the mode of action, IGRs had been recently grouped in chitin synthesis inhibitors (CSIs) and substances that interfere with the action of insect hormones (i.e., juvenile hormone analogues and ecdysteroids) [31]. IGRs can control many types of insects including fleas, cockroaches, and mosquitos even though they are not so fatal for adult insects [31]. Although low in toxicity to humans, they prevent reproduction, egg-hatch, and molting from one stage to the next in the young insects, while mixing them with other insecticides is able to kill even the adult insects [31].

### 2.3. GMO Products

These substances are produced through genetically modified organisms (GMOs). The genetic material is incorporated into the plant, which is then used as a source to produce pesticidal compounds, also referred to as plant-incorporated protectants (PIPs). Cry proteins are, by far the first-generation insecticidal PIPs that were introduced into the GM crops containing transgenes from the soil bacterium Bt. [30]. PIPs also demand the state of the research necessary for the ongoing environmental fate assessment of these molecules, primarily the RNAi-based PIPs [30,32] that would be discussed in a separate section.

## 3. Mode of Action of Biopesticides

Biopesticides act in a variety of ways on microorganisms depending on their type and nature. A few mechanisms through which biopesticides attack or kill pathogens are listed as follows [8].

### 3.1. Microbial Biopesticides

Fungicides and bactericides. These biopesticides generally inhibit or disrupt the process of translation and thus protein synthesis in numerous ways, including through binding of 50S ribosomes in prokaryotes, to prevent the transfer of peptides and inhibit chain elongation (such as blasticidin) [32,33]. Sometimes they interfere with the binding of aminoacyl tRNA to 30S and 70S ribosomal subunit complexes and inhibit translation (such as kasugamycin) [34]. In the case of streptomycin and mildiomycin, binding with the 30S ribosomal subunit causes abnormal synthesis of protein (nonfunctional) and blocks the activity of peptidyltransferase, respectively [35,36].

They can also disrupt plasma membrane permeability and cause leakage of substances (amino acids and electrolytes), thereby causing cell death (such as natamycin), and can inhibit chitin synthase activity (polyoxins) and inhibit trehalase, preventing the formation of glucose (validamycin) [31].

Insecticides upon reaching nerve endings, release gamma-aminobutyric acid (GABA), which causes GABA-gated Cl-ion channels to open, thus working by hyperpolarising the nerve membrane potential and blocking the electrical nerve conduction (avermectins and emamectin) [35,36]. Polynactins can cause leakage of potassium ions from mitochondria [36].

Herbicides inhibit phosphorylation in plants by blocking glutamine synthase, which causes an increase in ammonia (bilanafos) [36].

### 3.2. Biochemical Pesticides

These pesticides are derived from plants. Plants have evolved and developed many compounds, which can help to combat pathogenic microorganisms during the course of infection and attack. These compounds include steroids, alkaloids, phenylpropanoids, phenolics, terpenoids, and nitrogenated compounds. For instance, nicotine was the first insecticide obtained from tobacco leaves in the 17th-century that used to kill plum beetles [37,38]. Nicotine in tobacco is toxic to most herbivore insects and pesticides derived from them have been regarded as ‘green pesticides’ with high activity and low toxicity [38]. Duan et al. [38] have mentioned tobacco to be containing some useful ingredients, such as solanesol and nicotine, which exhibit potent inhibitory activity against *Staphylococcus aureus*, *Bacillus subtilis*, and *Micrococcus lysodeikticus*. Insecticides, such as azadirachtin and nicotine, function by either disrupting respiratory enzymes or inhibiting insect growth regulators, or by binding to sodium channels [39], while microbicides impair metabolic function and disrupt the integrity of plasma membrane and inhibit conidial formation [40].

### 3.3. GMO-Based Biopesticides

These are produced when genes are transferred into a plant, which allows it to produce compounds, such as Bt toxin, that can be used to combat pests. The delta endotoxins produced by the bacterium *B. thuringiensis* are broken down into smaller toxins in the insect gut by the action of proteases, which then bind to receptors in the midgut, causing cell expansion, rupture, and ion leakage leading to cell death [40].

## 4. Biopesticides from Algal and Cyanobacterial Sources

Microalgae can be used as an alternative technology to increase productivity in sustainable agricultural systems. A number of microalgae strains produce biologically active compounds that include antimicrobial compounds with the potential to act as biopesticides [40,41]. The biomass (extracts) can be applied as an alternative to chemical pesticides [40,42] since it can enhance plant growth and protect agricultural crops [42]. The filamentous cyanobacterium *Nostoc piscinale* and two single-celled green algae, *Chlamydopodium fusiforme* and *Chlorella vulgaris* are reported to have biopesticide activity against certain pathogens (Table 1). Some important microalgae have been exploited for their beneficial biopesticide activity in the cultivation of spices [43].

The use of chemical insecticides can result in numerous undesirable effects, including (i) killing of beneficial and nontargeted organisms and sometimes resurgence; (ii) rapid multiplication of secondary pests; (iii) development of pesticide resistance; (iv) contamination of the environment/ecosystem; (v) accumulation of pesticide residues in food materials; (vi) causing imbalanced ecological processes, such as pollination (pollinators affected by pesticides) and harm to living beings; (vii) carcinogenic and teratogenic effects in nature; and (viii) causing imbalances in hormone systems [8,73,74,75].

Several microorganisms have been explored for their potential in developing biopesticides. Microalgae have proved to be an excellent source owing to their advantages over traditional chemical pesticides. They produce a plethora of compounds with stimulating activities, including biomass and compounds, which can be used in the preparation of biopesticides, thereby enhancing crop protection [41]. Microalgae can be produced using wastewater, as they require nitrogen, phosphorus, and carbon and ammonium, which are abundant in wastewater, thus representing a nitrogen source. *Chlorella vulgaris* is generally used in the treatment of wastewater and is able to tolerate ammonium levels effectively. Ranglova et al. [41] assayed the efficacy of *C. vulgaris* against several phytopathogens, such as *Rhizoctonia solani*, *Fusarium oxysporum*, *Phytophthora capsica*, *Pythium ultimum*, *Clavibacter michiganensis*, *Xanthomonas campestris*, *Pseudomonas syringae*, and *Pectobacterium carotovorum*, while observing its antibacterial and antifungal activity, which were higher when cultivated in wastewater [41].

Gonçalves [3] argued that rice fields heavily sprayed with synthetic fertilisers to promote better productivity and yield left many detrimental effects on the environment and beneficial soil microflora, including decreased efficiency of fertiliser utilisation by the promotion of rice diseases, inhibition of microbiological nitrogen fixation, and increased nonpoint source pollution; importantly, they were also not cost effective. Furthermore, he added that in developing green rice, *Anabaena variabilis* could be a potent biofertiliser and biopesticide [3].

## 5. Biopesticide Activity from RNAi-Based Treatments

RNA interference technology is being used in the production of biopesticides due to the increased sensitivity towards pests and pathogens. Many transgenic crops (maize, soybean, and cotton) have been developed for resistance against particular pests [32]. Due to the limited consumption of genetically modified crops, RNA interference (RNAi) can be used as an alternative to overcome this problem. Studies carried out by Ratcliff et al. [76] and Ruiz et al. [77] demonstrated that transgenes had a significant impact on the functioning of plants upon viral infection through an RNAi mechanism. Similarly, Wang et al. [78] produced a barley crop completely resistant to barley yellow dwarf virus [76,77,78].

The mechanism of RNAi includes the expression of transgene dsRNA, which induces virus resistance and gene silencing in plants. Guide RNAs are formed as intermediaries; these are around 25 nt long and guide target RNAs for their degradation [79,80,81]. Dalmay et al. [81] reported that the process involves the use of RNA-dependent RNA polymerase RDR6 to generate double-stranded RNA (dsRNA) from target transcripts in plants, leading to the formation of small interfering RNA (siRNA) which, in turn, has silencing potential [81]. The RNase III domain-containing enzyme responsible for dsRNA cleavage, as observed in *Drosophila*, is called Dicer (also seen in plants and fungi) [82,83]. Following this, RNA-induced silencing complex (RISC)—a member of the conserved Argonaute family—is recruited, which mediates the cleavage of the target transcript [84,85], thus conferring resistance to the host [86].

RNAi technology has been used as a promising tool to overcome the ill effects of pests and pathogens. An RNAi method for oral application was developed by Baum et al. [85] using an artificial diet or transgenic maize against western corn rootworm (*Diabrotica virgifera*) to target V-ATPase subunits and alpha-tubulin [85]. Similarly, research conducted by Mao et al. showed the induction of growth defects in *Helicoverpa armigera*, the cotton bollworm, when given plant leaf material expressing a dsRNA specific to a cytochrome P450 gene [87]. The first commercial, genetically modified variety showing the expression of dsRNA against an insect pest was developed in 2017 when Monsanto and Dow approved SmartStax PRO maize containing dsRNA against the western corn rootworm Snf7 gene [88]. Similarly, apple and potato expressing dsRNAs were approved for regulation of endogenous gene expression for quality enhancement [88,89]. Apart from insects and viruses, the mechanism of RNAi-mediated silencing has been used to control other plant pests and pathogens, including bacteria such as *Agrobacterium*, fungi such as powdery mildew, and root-knot nematodes [86,90]. The US environmental protection agency (EPA) approved the first PIP called SmartStax Pro in June 2017 that will help US farmers control corn rootworm, a devastating corn pest that has developed resistance to several other pesticides [91].

## 6. Bacteria-Based Biopesticide

Pesticides formulated using microorganisms and their products are highly effective, species specific, and eco-friendly, leading to acceptance of their use in pest management strategies worldwide [8,9,10,17]. Given their significance as stated [8,9,10,17], there is enough scope for further development in their marketing and profitability for the manufacturing industry.

The bacteria that are used as biopesticides can be divided into four categories [92], namely, crystalliferous spore formers (such as *Bacillus thuringiensis*), obligate pathogens (such as *B. popilliae*), potential pathogens (such as *Serratia marcescens*), and facultative pathogens (such as *Pseudomonas aeruginosa*). Of these, spore-forming bacteria are the most widely sought after for commercial use. The most commonly used bacteria, *B. thuringiensis* and *B. sphaericus*, are highly specific, safe, and effective organisms for insect control [92].

The Cry family of crystalline proteins are produced by *B. thuringiensis* in the parasporal crystals and encoded by the *cry* genes. The Cry proteins are globular molecules (65–145 kDa, depending on the strain) with three structural domains connected by single linkers. The Cry proteins belong to a single family that contains about 50 subgroups [92]. Further details of the Cry protein and its mechanism of action have been elaborately discussed by Koul [44]. Finally, pests are killed by lethal septicaemia and starvation. An example of a *Bacillus sphaericus*-based product has been known to contain a binary mosquito larvicidal toxin comprising BinB (51.4 kDa) and BinA (41.9 kDa), which is commonly used for mosquito control [44].

## 7. Biopesticides from Arbuscular Mycorrhizal Fungi (AMF)

Arbuscular mycorrhizal fungi (AMF) play a crucial role in enhancing the growth and yield of crops [93,94]. They enhance the resistance of crops against pathogens by raising their defences. The composition of AMF changes and its presence decreases depending upon the soil type and crop, as well as the application of fertilisers and tillage [95,96]. Plants have evolved many direct and indirect mechanisms to overcome herbivory; for example, they produce chemicals such as nicotine, gossypol, and many other such compounds, which can prevent herbivores from feeding on them. It has been observed that AMF colonisation on crop plants is extremely helpful in providing a good defensive ability in hosts by altering the gene expression patterns and directly or indirectly changing the nutritional status of crops [97,98]. Some examples of the utility of AMF application in plant biocontrol are mentioned in Table 1. More research needs to be carried out in this area.

## 8. Nanobiopesticides

The concept of ‘nano’ in biopesticides has revolutionised the field due to the size, structure, and nature of substances, which are formed in a size range of 1–100 nm. These small biologically active particles can prevent the growth of pathogens by either destroying or repelling them [97,98,99]. Nanoencapsulation, nanocontainers, and nanocages, because of their property of degradability, increase the stability and efficacy of pest control, and lower amounts are used when delivering nanobiopesticides [99]. The damages caused by the phytopathogens can also be overcome by the application of nanobiopesticides, primarily the metallic nanoparticles (NPs) of zinc, gold, silver, nickel, and titanium owing to their inherent antimicrobial properties. These have some added advantages over other biopesticides because of their increased solubilisation abilities and target-oriented delivery of the compound with enhanced efficiency. Bacterial, fungal, and plant extracts are used for the synthesis of NPs. It has been shown that silver nanobiopesticides (AgNPs) can be synthesised using marine organisms such as *Sargassum muticum*, *Mesocyclops longisetus*, and *Caulerpa scalpelliformis* [71]. The benefit of the use of microorganisms in the preparations of NPs is that microorganisms can withstand high concentrations of metals over plants and also their rate of production and management is much easier, as compared to the plants. Needless to stress here that microorganisms being very tiny, have better penetration ability than plants. Narware et al. [71] have mentioned a number of microorganism-derived NPs that are very useful in pest control (Table 1).

Bioherbicides have also been used in the formulations of nanobioherbicides. The efficacy of metabolites of *Photorhabdus luminescence*, an endosymbiotic bacterium of the *Heterorhabditis indica*, entomopathogenic and parasitic nematodes, are controlled [98]. Similarly, nanofungicides have also been prepared to control various pathogenic fungi which include *Bipolaris sorokiniana*, *Fusarium* sp., *Alternaria alternata*, and many others through AgNPs and *Magnaporthe grisea* and *B. sorokiniana* using metal nanoparticles. Apart from their ability of being readily soluble, the nanofungicides are very economical, eco-friendly, and safe [98].

## 9. Biopesticides from Aquatic Plants

Duckweed (*Lemna minor*), muskgrass (*Chara* spp.), water hyacinth (*Eichhornia crassipes*), hydrilla (*Hydrilla verticillate*), water lettuce (*Pistia stratiotes*), and filamentous algae (*Lyngbya wollei*) are some common aquatic plants. It is observed that some plants produce allelopathic compounds which have the potential to prevent the growth, germination, survival, and reproduction of surrounding organisms. Neem (*Azadirachta indica*) extract kills many insects, while *Eichhornia crassipes* has the ability to inhibit the growth of *Spodoptera litura*, a lepidopteran pest [100,101,102,103]. Similarly, *Chenopodium album* is inhibited by the presence of duckweed and water lettuce [100]. These examples illustrate that similar plants (or weeds) and their allelopathic chemicals have highly potent inhibitory properties against the pathogens and hence can be substituted for conventional chemical pesticides [103].

## 10. Merits of Biopesticides over Chemical Pesticides

Biopesticides have several merits over conventional chemical pesticides. They are environmentally friendly, target specific, and not deleterious to nontarget organisms and hence potent enough to replace synthetic pesticides for pest management [46]. Table 2 provides an overview of the disadvantages of using conventional chemical pesticides instead of biopesticides.

In recent years, the use of biopesticides is gaining momentum because they can be efficiently used in sustainable agricultural practices [2,3]. Biopesticides are highly effective in small amounts and decompose quickly without leaving problematic residues and hence can reduce the use of conventional pesticides as an integral component of IPM programs [102]. However, despite the merits of using biopesticides, their use has not been as widespread as expected, for the following reasons:

1. High cost of pesticide production due to the costs involved in screening, developing, and getting regulatory clearance for new biological agents;
2. Short shelf life due to the sensitivity of biopesticides to fluctuations in temperature and humidity;
3. Limited field efficacy due to climatic/regional variations in temperature, humidity, soil conditions, etc.;
4. Due to the high specificity of the biopesticides, i.e., they are only effective against target pathogens and pests, farmers are disinterested in them. They need to use multiple biological agents to control different pathogens and pests in the field. These agents are confusing, costly, and cumbersome, and are also not available for every pest or pathogen.

## 11. Commercial Exploitation of Biopesticides

Currently, a majority (about 90%) of microbial biopesticides on the market are derived from a single bacterium, *Bacillus thuringiensis* or Bt. Biopesticides make up a small share of the crop protection market, with a value of about USD 3 billion worldwide, accounting for just 5% of the total market [102]. In the United States market, more than 200 products are available, while the European Union market has only 60 analogues [43]. Biopesticide use at a global scale is increasing by almost 10% every year [43]. However, these pesticides are going to contribute noticeably to their global market consumption needs, which are to increase further in the future by substituting them for and thus reducing the over-reliance on chemical pesticides. Biopesticides are assessed in the EU by the same regulations used for the assessment of synthetic active substances, which require the addition of several new provisions in the current legislation, and the preparation of new guidelines facilitates the registration of prospective biopesticide products [78]. It is assumed that there are fewer active substances of biopesticides registered in the EU than in the USA, India, Brazil, or China [104]. It is expected that the use of biopesticides will be on par with synthetics by the early 2050s, but major uncertainties regarding the rates of uptake, especially in areas such as Africa and Southeast Asia, account for most of the flexibility in such projections [102].

## 12. Conclusions

The application of biofertilisers consisting of bacteria, cyanobacteria, or fungi can improve and restore the fertility of the soil and ensure sustainable agricultural production using green technology. Using microorganisms and microalgae as biopesticides can reduce the demand for energy and consumption of synthetic fertilisers and restore the efficiency of agroecosystems and wastelands. These organisms, when combined with the use of biotechnical innovations such as RNAi technology, can play a significant role in the production of secondary metabolites, biofertilisers, bioenergy, and bioprocessed products that would be also useful in pest control. RNAi-based biopesticides have gained enough momentum in recent years as a narrow-spectrum alternative to chemical-based control measures for specific and accurate targeting of pests and pathogens. In this regard, the use of bioinformatics-based dsRNA selection for effective RNAi design, coupled with adequate experimental testing, will likely eliminate the adverse impacts of RNAi-based biopesticides [86].

Considerable research on biological control agents, including biopesticides, is required for the development of the biopesticide market in the future. Scientists from diverse research institutes around the world are engaged in enormous research efforts in the field, but very few complete and systematic reports are available. Here, the utmost collaboration among enterprises and research institutes is needed, without which a scenario whereby biopesticides completely replace chemical pesticides seems impossible. In the current scenario, the agricultural sector needs to rely on both biopesticides and chemical pesticides. However, speeding up the practical application of laboratory results should facilitate large-scale industrial development. The inflow of biopesticides, however, has considerably reduced the use of synthetic chemicals because of stringent regulations [102]. Many substances have been researched to demonstrate their utility as biopesticides (Table 1), but extensive field research is required in order to assess their efficacy for precise pest problems under diverse cropping systems.

Farmers and society at large should benefit from the mixed and judicious use of both conventional chemical pesticides and biopesticides, while it is imperative to emphasise the research in the area of biopesticides for reaping greater benefits from it in the future.

## Figures and Tables

**Table 1:** A broad description of some common biopesticides, their types, sources, and target crops with the authors who published such reports.

Source	Type	Organism	Pest Type	Target Crop	Reference(s)
Bacteria	Insecticide	*Bacillus thuringiensis* var *kurstaki* *B. thuringiensis* var *tenebrionis*	caterpillars, fungi (*Botrytis*) Elm Leaf Beetle, Alfalfa weevil	vegetables, fruits, ornamentals, cereals Potato	Koul [44]; Bravo et al. [45] Saberi et al. [46]
	fungicide	*Bacillus subtilis*	*Botrytis* spp.	vegetables, fruits, and ornamentals	Koul [44]; Bravo et al. [45]
Fungi	insecticide	*Beauveria bassiana*	Whitefly	protected edible and ornamental plant production	McGuire and Northfield [47]
	fungicide	*Coniothyrium minitans* *Trichoderma harzianum*	*Sclerotinia* spp. *S sclerotiorum*.	outdoor edible and nonedible crops and protected crops Starwberry crops	Gams et al. [48] Dolatabadi et al. [49]
	herbicide	*Chondrostereum purpureum*	cut stumps of hardwood trees and shrubs	Forestry	Bailey [50]
	nematicide	*Paecilomyces lilacinus*	plant-parasitic nematodes in soil	vegetables, soft fruit, citrus, ornamentals, tobacco and turf	Moreno-Gavíra et al. [51]
Virus	insecticide	*Cydia pomonella* granulovirus	codling moth	apples and pears	Kadoić Balaško et al. [52]
Oomycetes	herbicide	*Phytophthora palmivora*	*Morenia orderata*	citrus crops	Lala et al. [53]
Neem (*Azadirachta indica)*	insecticide	Azadirachtin	aphids, scale, thrips, whitefly, leafhoppers, weevils	vegetables, fruits, herbs, and ornamental crops	Chaudhary et al. [54]
Plant extracts	fungicide	*Reynoutria sachalinensis* (giant knotweed) extract	powdery mildew, downy mildew, *Botrytis*, late blight, citrus canker	protected ornamental and edible crops	Marrone [55]
	herbicide	Plant essential oils	Ragwort, many arthropods	Grassland	Isman [56]
	nematicide	*Quillaja saponaria*	plant parasitic nematodes	vineyards, orchards, field crops, ornamentals and turf	Guerra and Sepúlveda [57]
*Talaromyces flavus*; *Clitoria ternatea* (butterfly pea); *Trichoderma harzianum*; *Bacillus thuringiensis* var. *tenebrionis*; *Lactobacillus casei* fermentation products	biopesticides		*Glomerella cingulata* and *Colletotrichum acutatum*; *Helicoverpa* spp.; *Fusarium oxysporum Agelastica alni*; *Spodoptera litura*, *Helicoverpa armigera*, *Aphis gossypii*; *Xanthomonas fragariae*; *Spodoptera littoralis* and others	Strawberry, Cotton, Gladiolus hybrids, alder leaf, and hazelnut, other economically important plants and trees	Ishikawa [58]; Mensah et al. [59].; Kirk and Schafer [60] Eski et al. [61].; Dubois et al. [62]; Pavela et al. [63]; El-Abbassi et al. [64]
Semiochemical	attractant	Citronellol	tetranychid mites	apples, cucurbits, grapes, hops, nuts, pears, stone fruit, nursery, and ornamental crops	Mauchline et al. [65]; Mossa et al. [66]
	attractant	Multi-component sex pheromone, such as (E,E)-8,10-dodecadien-1-ol	codling moth	Fruits, such as apples and pears	El-Sayed et al. [67]
Arbuscular Mycorrhizal Fungi	Mutual inhabitant in the roots	Fungi	*Fusarium verticillioides;* pathogens affecting below ground plant organs	*Zea mays*	Olowe et al. [68]; Bharadwaj and Sharma [69]; Mukerji and Ciancio, [70]
Microalgae	Filamentous cyanobacterium; Single-celled green algae	*Nostoc piscinale; Chlamydopodium fusiforme*; *Chlorella vulgaris*	-	-	Ranglova et al. [41]
		*Anabaena laxa* and *Calothrix elenkinii*	Increase in fungicidal activity	Coriander, cumin, and fennel	Kumar et al. [43]
Nanobiopesticide	Silver nanobiopesticide	None	*Alternaria alternata*, *A. solani*	Alternaria leaf blight and leaf spot diseases in tomato, pepper, and potato	Narware et al. [71]
	*Sargassum muticum* derived NPs	None	*Ariadne merione*, a Lepidopteran pest	-	Narware et al. [71]; Rodrigues et al. [72]
	*Caulerpa scalpelliformis* and *Mesocyclops longisetus*-derived NPs	None	*Culex quinquefasciatus*	-	Narware et al. [71]

**Table 2:** The various disadvantages of conventional chemical pesticides over biopesticides.

Conventional Chemical Pesticides	Biopesticides
Synthesised or produced from artificial/chemicals	Use naturally occurring compounds derived from living organisms for the production
They cause environmental pollution and are not eco-friendly	They do not cause environmental harm
Harmful to nontarget organisms	Do not cause harm to nontarget organisms
Cost ineffective	Cost efficient and cheaper, compared to chemical fertilisers
Microorganisms develop resistance gradually as the application increases	Pests do not develop resistance
High market value	Not preferred in the market
Contaminate water and soil	Cannot contaminate water sources
Lead to bioaccumulation	Do not lead to bioaccumulation

## Data Availability

Not Applicable.
